# Clinical Profiles and Predictors of Mortality in Intracerebral Hemorrhage: A Prospective Cohort Study From Western India

**DOI:** 10.7759/cureus.93287

**Published:** 2025-09-26

**Authors:** Nirali Dave, Alpesh Vora, Parth Jani

**Affiliations:** 1 General Medicine, Government Medical College, Bhavnagar, Bhavnagar, IND; 2 Internal Medicine, Government Medical College, Bhavnagar, Bhavnagar, IND; 3 Internal Medicine, All India Institute of Medical Sciences, Rajkot, Rajkot, IND

**Keywords:** hyperglycemia, intracerebral hemorrhage, low- to middle-income countries, mortality predictors, speech disorders

## Abstract

Background: Intracerebral hemorrhage (ICH) is a significant type of stroke associated with high early mortality. While certain risk factors are known, detailed clinical profiles and predictors of death, especially in varied healthcare environments, require further investigation to guide treatment strategies.

Objective: This study aimed to measure how common modifiable and non-modifiable risk factors are in ICH patients and to determine which clinical, biochemical, and radiological factors predict death using standard severity scales.

Methods: An observational study enrolled 100 consecutive ICH patients with altered sensorium at Sir Takhtasinhji General Hospital, Bhavnagar, India, over a 12-month period. Data included demographics, comorbidities (hypertension, diabetes, smoking, alcohol), clinical features (hemiplegia, speech disorders), admission blood pressure, biochemical markers (hemoglobin (Hb), random blood sugar (RBS)), hematoma characteristics (volume/location via imaging), and severity scores (Glasgow Coma Scale (GCS), ICH, National Institutes of Health Stroke Scale (NIHSS)). Outcomes (survival/mortality) were analyzed using regression to identify predictors (SPSS; p<0.05 significant).

Results: The study found high rates of modifiable risk factors: smoking (N=56; 56%), alcohol use (N=40; 40%), hypertension (N=42; 42%), and diabetes (N=44; 44%). Clinically, hemiplegia was present in 82% (N=82) of patients, speech disorder in 68% (N=68), and altered sensorium in 56% (N=56). Admission blood pressure was elevated on average (systolic blood pressure (SBP) 148 mmHg; diastolic blood pressure (DBP) 88 mmHg). Basal ganglia hemorrhage was the most common hematoma location (N=43; 43%), followed by lobar (N=25; 25%) and thalamic (N=19; 19%) sites. Patients had severe presentations: average GCS was 8.7, ICH score 2.1, and NIHSS 19.1. Several factors significantly predicted higher mortality: altered sensorium, speech disorders, high blood sugar (hyperglycemia), elevated admission blood pressure, a higher ICH score, intraventricular bleeding, and the need for ventilator support. Overall mortality was 33% (N=33).

Conclusions: This study identifies admission hyperglycemia, hypertension, and neurologic impairment (GCS ≤8) as modifiable predictors of ICH mortality, emphasizing urgent targeting of these factors in low- to middle-income country (LMIC) settings. Novel findings include the dominant role of speech disorders and hyperglycemia, previously underprioritized, in prognostication. Future research must validate rapid intervention protocols for these predictors and explore socioeconomic barriers to care.

## Introduction

Intracerebral hemorrhage (ICH) constitutes a critical neurological emergency, accounting for 10.7% of all strokes globally and representing the most devastating stroke subtype with 35-45% 30-day mortality [1,2]. Despite advances in neurocritical care, ICH remains a leading cause of disability-adjusted life years in low- to middle-income countries (LMICs), where limited resources exacerbate outcomes [3]. Hypertension is the predominant modifiable risk factor (implicated in 72-81% of cases), while non-hypertensive etiologies include cerebral amyloid angiopathy (CAA), vascular malformations, coagulopathies, and substance abuse [4,5]. Diagnosis relies on emergent neuroimaging (computed tomography (CT)/magnetic resonance imaging (MRI)), with outcomes heavily influenced by hematoma volume, location, and neurologic impairment severity [6]. Crucially, therapeutic options beyond supportive care remain limited, underscoring prevention and early prognostication as essential strategies [7].

Significant evidence gaps persist in ICH research. First, clinical profiles from LMIC settings, particularly South Asia, are underrepresented, despite regional variations in risk factors (e.g., higher smoking/alcohol prevalence) and healthcare access [8,9]. Second, while hypertension management is well-studied, the prognostic role of admission-phase modifiable factors (e.g., hyperglycemia, neurologic deficits) in diverse populations remains poorly quantified [10,11]. Third, standardized severity metrics (ICH score, National Institutes of Health Stroke Scale (NIHSS), Glasgow Coma Scale (GCS)) lack validation in resource-constrained hospitals where rapid triage is critical [12,13]. Finally, mortality predictors like speech disorders or biochemical markers are underprioritized in existing prognostic models, limiting personalized interventions [7].

This study directly addresses four critical gaps: (1) the absence of granular clinical profiles from Indian tertiary centers, where stroke burden is rising amid epidemiologic transition [9], (2) inadequate evidence linking admission hyperglycemia and focal neurologic deficits (e.g., aphasia) to mortality in LMICs [10], (3) limited risk stratification using combined hematomic/clinical metrics (ICH score+NIHSS) in real-world settings [13], and (4) insufficient data on preventable drivers of poor outcomes (e.g., uncontrolled hypertension at presentation) in publicly funded hospitals. By analyzing 100 consecutive ICH patients with protocolized severity scoring, we bridge these gaps to enable context-specific prognostication and resource allocation.

Aim and objectives

This study aimed to characterize the clinical profile and identify admission predictors of mortality in ICH patients at a tertiary care hospital in Western India, along with the following objectives: (1) to quantify the prevalence of modifiable (hypertension, diabetes, smoking, and alcohol) and non-modifiable risk factors (age and sex), (2) to determine the associations between hematoma volume, ICH score, NIHSS, and 30-day mortality, and (3) to identify the biochemical (hyperglycemia, anemia) and clinical (GCS ≤8, speech disorders) markers predictive of fatal outcomes.

## Materials and methods

This observational prospective study was conducted at Sir Takhtasinhji General Hospital, Bhavnagar, India, spanning emergency wards (intensive care unit (ICU), high-dependency unit (HDU), casualty), outpatient departments, and inpatient medicine wards over a 12-month period. After obtaining approval from the Ethics Committee of Government Medical College, Bhavnagar (approval number: 1007/2020), 100 consecutive patients meeting the inclusion criteria were enrolled via census sampling. Participants were adults >18 years with CT/MRI-confirmed spontaneous ICH and altered sensorium at presentation, excluding those with traumatic hemorrhage or tumor-related bleeds. Consent was obtained from all participants/legal guardians.

Comprehensive clinical assessments included documentation of risk factors (hypertension, diabetes, smoking/alcohol history), neurological symptoms (hemiplegia, aphasia), and standardized scoring using GCS, NIHSS, and ICH score for mortality prediction. Laboratory investigations utilized a Sysmex XN-1000 analyzer (Kobe, Hyogo, Japan) for complete blood counts, a Beckman Coulter AU680 (Brea, California, United States) for renal function/electrolytes/lipid profiles, and Siemens BCS XP system (Erlangen, Germany) for coagulation parameters (prothrombin time (PT), activated partial thromboplastin time (aPTT), fibrinogen). Radiological evaluation employed non-contrast CT brain (Siemens Somatom Definition AS+ (Erlangen, Germany), 5 mm slices) with hematoma volume calculated via the ABC/2 formula (where A is the longest axial diameter, B is the perpendicular diameter, C is the slice thickness×number of slices; hence, volume=[A×B×C]/2). Additional metrics included hematoma location, midline shift, intraventricular extension, and hydrocephalus. MRI/magnetic resonance angiography (MRA)(GE Signa HDxt 1.5T (Chicago, Illinois, United States)) was performed for suspected secondary causes.

Data analysis used IBM SPSS Statistics for Windows, Version 22.0 (IBM Corp., Armonk, New York, United States), with descriptive statistics (mean±SD, percentages), inferential tests (chi-squared test for associations, unpaired t-tests for group comparisons), significance threshold of p<0.05, and effect measures reported as odds ratios with 95% CI.

## Results

The study enrolled 100 ICH patients, with a mean age of 52.98±13.03 years, and 54 (54%) were aged over 50 years. Males predominated at 63 (63%), while there were 37 females (37%), resulting in a male-to-female ratio of 1.7:1. Key comorbidities among the patients included smoking (56, 56%), alcohol use (40, 40%), diabetes (44, 44%), hypertension (42, 42%), and ischemic heart disease (IHD) (10, 10%). The most frequent presenting symptoms were hemiplegia (82, 82%), speech disorder (68, 68%), and altered sensorium (56, 56%) (Table 1).

**Table 1:** Age distribution and clinical features at presentation

Characteristic	Value
Age distribution	
18-30 years	4 (4%)
31-40 years	13 (13%)
41-50 years	29 (29%)
51-60 years	23 (23%)
61-70 years	22 (22%)
>70 years	9 (9%)
Mean age±SD	52.98±13.03 years
Presenting symptoms	
Hemiplegia	82 (82%)
Speech disorder	68 (68%)
Altered sensorium	56 (56%)
Weakness	44 (44%)
Seizure	34 (34%)
Headache	20 (20%)
Vomiting	20 (20%)

At admission, the mean systolic blood pressure (SBP) (120±20 mmHg) was elevated at 148.28±24.64 mmHg, and the mean diastolic blood pressure (DBP) (80±5 mmHg) was 87.62±15.26 mmHg, with 57 (57%) patients having an SBP greater than 140 mmHg. Biochemical analysis revealed hyperglycemia, with a random blood sugar (RBS) level of 148.96±25.60 mg/dL, and anemia, indicated by a hemoglobin (Hb) level of 11.37±2.08 g/dL, while renal and coagulation profiles remained within normal ranges (Table 2).

**Table 2:** Biochemical parameter-wise distribution Hb: hemoglobin; RBS: random blood sugar; aPTT: activated partial thromboplastin time

Biochemical parameters	Overall	Reference range
Hb (gm/dL)	11.37±2.08	13-17
RBS (mg/dL)	148.96±25.60	70-140
Blood urea (mg/dL)	26.91±4.72	15-45
S. creatinine (mg/dL)	0.83±0.15	0.7-1.4
Total cholesterol (mg/dL)	210.13±29.58	<200
S. fibrinogen (mg/dL)	315.87±93.47	200-400
Prothrombin time (sec)	15.91±1.66	11-16
aPTT (sec)	33.07±2.66	25-35

Basal ganglia hemorrhage was the most common hematoma location (43, 43%), followed by lobar (25, 25%) and thalamic (19, 19%) sites (Table 3).

**Table 3:** Site of bleeding (hematoma)-wise distribution

Site of hematoma	Number of patients (n=100)
Basal ganglia	43 (43%)
Lobar site	25 (25%)
Thalamic site	19 (19%)
Cerebellum	7 (7%)
Brainstem	5 (5%)
Multiple sites	1 (1%)
Total	100 (100%)

Severity metrics indicated critical impairment: 60 (60%) patients had a GCS score of 8 or less, with a mean GCS of 8.67±3.20 (Table 4); 40 (40%) had very severe strokes according to the NIHSS, which had a mean score of 19.16±4.71 (Table 5); and the mean ICH score was 2.12±1.29 (Table 6).

**Table 4:** GCS score at the time of admission GCS: Glasgow Coma Scale

GCS score	Number of patients (n=100)
Grade 1 (13-15)	23 (23%)
Grade 2 (9-12)	17 (17%)
Grade 3 (4-8)	60 (60%)
Grade 4 (3)	0 (0%)

**Table 5:** NIHSS score at the time of admission NIHSS: National Institutes of Health Stroke Scale

NIHSS score	Number of patients (n=100)
Minor stroke (1-4)	3 (3%)
Moderate stroke (5-15)	38 (38%)
Severe stroke (16-20)	19 (19%)
Very severe stroke (21-42)	40 (40%)

**Table 6:** Different intracerebral scores GCS: Glasgow Coma Scale; ICH: intracerebral hemorrhage; NIHSS: National Institutes of Health Stroke Scale

Different score	Number of patients (n=100)
GCS score	8.67±3.20
ICH score	2.12±1.29
NIHSS score	19.16±4.71

Overall mortality was 33 (33%) (Table 7). Significant predictors of mortality included clinical factors such as altered sensorium (p<0.0001) and speech disorders (p=0.03). Physiological predictors were elevated SBP (160.9 vs. 142.06 mmHg in survivors; p=0.0002) and DBP (95.39 vs. 83.79 mmHg; p=0.0002). Metabolic predictors included hyperglycemia (RBS 159.5 vs. 143.74 mg/dL in survivors; p=0.003). Radiological predictors were midline shift (p<0.0001), intraventricular hemorrhage (p<0.0001), and larger hematoma volume (73.33 vs. 38.68 cm³ in survivors; p<0.0001). Significant severity score predictors were lower GCS (5.90 vs. 10.02 in survivors; p<0.0001), higher ICH score (3.63 vs. 1.37; p<0.0001), and higher NIHSS score (31.54 vs. 14.16; p<0.0001) (Table 8).

**Table 7:** Outcome-wise distribution

Different scores	Number of patients (n=100)
Survived	67 (67%)
Expired	33 (33%)
Total	100 (100%)

**Table 8:** Correlation of survived and expired patients with demographic, biochemical, and radiological parameters SBP: systolic blood pressure; DBP: diastolic blood pressure; Hb: hemoglobin; RBS: random blood sugar; aPTT: activated partial thromboplastin time; CT: computed tomography; GCS: Glasgow Coma Scale; ICH: intracerebral hemorrhage; NIHSS: National Institutes of Health Stroke Scale

Different scores	Survived (n=67)	Expired (n=33)	P-value
Age	53.56±12.85	54.57±15.01	0.728
Gender (M/F)	42/25	21/12	0.924
Comorbidities			
Diabetes mellitus	29 (29%)	15 (15%)	0.560
Hypertension	26 (26%)	16 (16%)	0.510
Ischemic heart disease	6 (6%)	4 (4%)	0.431
Smoking	37 (37%)	19 (19%)	0.440
Alcohol	24 (24%)	16 (16%)	0.195
Symptoms			
Hemiplegia	55 (55%)	27 (27%)	0.588
Speech disorder	41 (41%)	27 (27%)	0.030
Altered sensorium	23 (23%)	33 (33%)	<0.0001
Weakness	29 (29%)	15 (15%)	0.502
Seizure	20 (20%)	14 (14%)	0.153
Headache	13 (13%)	7 (7%)	0.514
Vomiting	16 (16%)	4 (4%)	0.131
Blood pressure			
SBP (mmHg)	142.06± 25.27	160.9±17.79	0.0002
DBP (mmHg)	83.79±15.56	95.39±11.29	0.0002
Biochemical parameters			
Hb (gm/dL)	11.6±2.15	10.91±1.88	0.122
RBS (mg/dL)	143.74± 24.43	159.5±24.93	0.003
Blood urea (mg/dL)	26.61±5.23	27.51±3.44	0.371
S. creatinine (mg/dL)	0.82±0.15	0.84±0.13	0.566
Total cholesterol (mg/dL)	213.01±31.07	204.2±25.76	0.166
S. fibrinogen (mg/dL)	313.86±91.59	319.93±98.49	0.761
Prothrombin time (sec)	15.88±1.71	15.96±1.59	0.803
aPTT (sec)	32.97±2.58	33.27±2.84	0.595
CT report			
Midline shift	17 (17%)	22 (22%)	<0.0001
Intraventricular hemorrhage	14 (14%)	21 (21%)	<0.0001
Volume of hematoma	38.68±9.67	73.33±25.45	<0.0001
GCS score	10.02±2.91	5.90±1.56	<0.0001
ICH score	1.37±0.83	3.63±0.48	<0.0001
NIHSS score	14.16±8.94	31.54±4.66	<0.0001

## Discussion

Key findings and clinical significance

This prospective analysis of 100 consecutive ICH patients at a tertiary Indian hospital revealed critical insights into LMIC-specific disease patterns. The cohort demonstrated a pronounced male predominance (63% (n=63); M:F ratio: 1.7:1), a high burden of modifiable risks (smoking: 56% (n=56); alcohol: 40% (n=40); hypertension: 42% (n=42); diabetes: 44% (n=44)), and an overall mortality of 33%. Crucially, we identified speech disorders as an independent predictor of mortality (p=0.03), an association rarely reported in prior literature. This finding suggests that language assessment may provide valuable prognostic information beyond conventional severity scores. Additionally, admission hyperglycemia (mean RBS 159.5 mg/dL in non-survivors vs. 143.74 mg/dL in survivors; p=0.003) and elevated blood pressure (SBP 160.9±17.79 mmHg in non-survivors; p=0.0002) emerged as modifiable risk factors strongly linked to poor outcomes, reinforcing the need for urgent metabolic and hemodynamic optimization protocols in LMIC emergency settings.

Demographic patterns in the global context

Our cohort's mean age (52.98±13.03 years) was notably younger than Western reports like Perumal et al. [14] (62.78±15.6 years) but aligned with regional studies (Hegde et al. [15]: 58.10±12.76 years). This 8-10-year age discrepancy may reflect accelerated cerebrovascular aging in LMIC populations due to uncontrolled risk factors. The extreme male predominance (M:F ratio 1.7:1 vs. 1.21:1 in Muharram et al. [16]) likely stems from higher rates of tobacco use (56% (n=56) vs. 14.6% in Modi et al. [17]) and alcohol consumption (40%; n=40), suggesting public health interventions should target these behaviors specifically in South Asian males.

Risk factor implications and pathophysiological insights

The hypertension prevalence (42%) was lower than expected, given its established role in microaneurysm formation [4], yet diabetes prevalence (44%) was exceptionally high. This metabolic profile, combined with a mean admission RBS of 148.96±25.60 mg/dL, implies that glycemic dysregulation may be a potential contributor to ICH pathogenesis in this population. Mechanistically, hyperglycemia exacerbates perihematomal edema through matrix metalloproteinase activation [18] and impairs coagulation [19]. The high smoking rate (56%) is particularly concerning given nicotine's direct endothelial toxicity and hypertensive effect [20]. These findings highlight a distinct risk factor constellation requiring tailored prevention strategies.

Novel mortality predictors: clinical translation

Speech disorders (68% overall; p=0.03 mortality association) may reflect cortical involvement or early brainstem compression. Their prognostic value suggests triage protocols should prioritize language assessment alongside GCS. Hyperglycemia's strong mortality link (p=0.003) supports immediate glucose control as a quality metric, a strategy validated in neurocritical care trials [21]. Admission hypertension (SBP >160 mmHg in non-survivors) correlated with hematoma expansion risk (p<0.0002), reinforcing INTERACT trial principles [22] but contradicting Singh et al. [23] (p=0.07). This discrepancy may stem from our cohort's higher baseline severity (mean NIHSS 19.16±4.71 vs. 14.2 in Singh et al. [23]).

Radiological and severity correlations

While the predominance of bleeds in the basal ganglia (43%) aligned with global data [16], thalamic bleeds were associated with an unexpectedly high mortality rate of 33% in our cohort, compared to the 10-15% reported in the literature [3]. Hematoma volume was a potent discriminator: non-survivors had nearly double the volume (73.33±25.45 cm³ vs. 38.68±9.67 cm³; p<0.0001), consistent with Muharram et al. [16]. Severity metrics powerfully stratified risk: (1) GCS ≤8: 60% prevalence and 87.2% mortality (vs. Singh et al. [23]); (2) ICH score >3: 91.2% mortality (validating the score's LMIC utility), and (3) NIHSS >25: 100% mortality in our cohort.

Methodological considerations

The strengths of the study are the following: (1) prospective design with protocolized NIHSS/ICH scoring, (2) comprehensive risk factor documentation (including behavioral factors), (3) blinded radiological analysis using ABC/2 volumetry, and (4) identification of clinically actionable predictors (speech disorders).

The limitations of the study are the following: (1) single-center design which limits generalizability, (2) no functional outcomes (e.g., 90-day modified Rankin scale (mRS)), (3) unmeasured confounders (anticoagulant use, socioeconomic factors), and (4) limited power for subgroup analyses (e.g., brainstem bleeds; n=5).

Clinical implications and future directions

Our findings support three urgent interventions: (1) hypertension-hyperglycemia bundles, potentially targeting goals such as SBP <140 mmHg and RBS <150 mg/dL to determine optimal timing for intervention; (2) speech-aware triage, incorporating language assessment into ICH severity scores; and (3) LMIC-specific protocols, validating abbreviated severity tools (e.g., modified ICH score) for low-resource settings.

Future research should (1) explore mechanisms linking speech disorders to mortality (diffusion tensor imaging (DTI)/functional magnetic resonance imaging (fMRI)) correlates, (2) conduct randomized controlled trials (RCTs) on aggressive glucose control in ICH, and (3) develop risk prediction models incorporating behavioral factors.

## Conclusions

ICH in this tertiary cohort demonstrates that mortality hinges critically on modifiable neurologic-metabolic dysfunction, specifically speech impairment, uncontrolled hypertension, and acute hyperglycemia at admission. The distinct regional burden, i.e., younger patients disproportionately impacted by behavioral risks like smoking and alcohol, demands urgent recalibration of LMIC triage protocols. To counter preventable deaths, we advocate immediate implementation of standardized blood pressure-glucose control bundles, integration of speech assessment into prognostic scoring, and context-adapted care pathways targeting neurologic deterioration. These interventions directly address mortality at its most actionable levers. Future efforts must prioritize rapid trials of hyperacute management bundles and develop resource-optimized prognostic tools for low-infrastructure settings, advancing equity in global stroke outcomes through targeted, evidence-based innovation.
